# Xerostomia in a Group of Turkish Patients Using an Online Survey During the COVID-19 Pandemic

**DOI:** 10.7759/cureus.64930

**Published:** 2024-07-19

**Authors:** Sevilay Yeğinoğlu, Gülden Ereş

**Affiliations:** 1 Periodontology, Karabük Oral and Dental Health Hospital, Ministry of Health, Karabük, TUR; 2 Periodontology, Faculty of Dentistry, Ankara University, Ankara, TUR

**Keywords:** xerostomia, covid-19, public health, oral health, xerostomia symptoms, xerostomia inventory

## Abstract

Background

Xerostomia is defined as the subjective feeling of dry mouth and affects millions of patients worldwide. Most studies are based on samples of the elderly in nursing homes. This study aimed to investigate the presence of xerostomia and the severity of self-reported xerostomia by sociodemographic variables and to evaluate xerostomia symptoms (self-reported halitosis, burning mouth, and mouth sores) in young adults.

Methodology

A questionnaire regarding sociodemographic data and the 11-item Xerostomia Inventory was delivered to patients aged 20-65 years who applied to the Ankara University Faculty of Dentistry for dental treatment before the COVID-19 pandemic. Statistical analyses were performed to determine the relationships between the presence of xerostomia and other variables such as age, gender, the presence of a systemic disease, medication use, smoking, alcohol consumption, and the use of removable prostheses.

Results

A total of 300 patients were included in the study. Xerostomia presence of 54.6% (164 patients) was identified. A significant relationship was found between age and xerostomia (p = 0.023; p = 0.001). The presence of xerostomia decreased as age increased. Xerostomia was more common in female patients (p = 0.028; p = 0.004). The presence of xerostomia was found to be high, not only in the elderly but also in younger adults.

Conclusions

This study sheds light on the current status, symptoms, and etiology of xerostomia presence in the young population in Turkey. Factors associated with xerostomia were age, female gender, and the number of cigarettes smoked per day. In this study, the high presence of xerostomia was due to smoking.

## Introduction

Salivary gland hypofunction (SGH) occurs when the salivary glands work under their capacity, resulting in hyposalivation with insufficient saliva or decreased salivary flow. The widely accepted clinical definition of xerostomia is a subjective feeling of dry mouth while SGH is the objective observation of reduced salivary flow. The term “dry mouth” has been used to describe both conditions [1]. Xerostomia may occur as a result of changes in the composition and flow of saliva, but the amount of saliva does not always decrease [2]. Some studies have shown that people may have hyposalivation but do not report signs of xerostomia. Similarly, people who report xerostomia may have a normal or high salivary flow [1,3,4].

Xerostomia is a common condition that causes problems such as difficulty in swallowing, loss of taste, and pain. It also increases the risk of tooth decay and infection in the mouth [5]. Patients with xerostomia may complain of a sense of burning mouth, halitosis, and mouth soreness [6]. It can lead to significant social problems that impair the ability or willingness to speak and may affect nutrition by inhibiting the sense of taste or causing insufficient chewing or swallowing, thereby exacerbating malnutrition [7]. This condition may severely affect the quality of life of patients.

Xerostomia is an important side effect of many commonly used drugs [8]. More than 400 drugs can cause salivary gland dysfunction, and it has been reported that 80% of the most commonly prescribed drugs cause hyposalivation [5,8,9]. Antidepressants, anxiolytic drugs, antihypertensives, diuretics, and antihistamines are the drugs that most commonly cause both xerostomia and hyposalivation [9].

The prevalence of xerostomia refers to the percentage of patients with symptoms of subjective dry mouth for a certain period in a certain population, and different prevalence values ranging from 0.9% to 64.8% have been reported by many studies conducted in different parts of the world [3,6,9-12]. The majority of these studies were conducted in Scandinavia. At the same time, most of the studies examined individuals aged 50 years or older. These studies describe the prevalence of xerostomia in the general population, and study populations ranged from 259 to 3,313 individuals. However, as xerostomia is a subjective sense of dry mouth experienced by a patient, it should only be measured through self-reported questionnaires [13].

In the literature, a single question has generally been used to evaluate the presence and symptoms of xerostomia, such as “Do you usually sense dryness in your mouth?” [10,14]. However, there have been attempts to develop new measurement tools, as this question limits questioning to only dry mouth among all xerostomia symptoms [1]. Xerostomia Inventory (XI) is a tool that has been scientifically validated and approved and is commonly used to evaluate the symptoms of patients with xerostomia [1,12,15-17].

The 11 items that constitute XI serve the purpose of examining both the experiential and behavioral aspects of the condition [18]. The original XI was developed in 1999 [1]. The validity and reliability analysis of the inventory in Turkish was performed, and it was reported to be valid and reliable [19].

Previous studies have mainly paid attention to xerostomia prevalence among older adults [5]. There are few epidemiological studies in the literature for the general population over the age of 18 [10,20,21]. Additionally, medication use is higher in people over 65 years of age, and the prevalence of xerostomia is found to be correspondingly high [22]. This study aimed to determine the presence and severity of self-reported xerostomia in patients who applied to the Ankara University Faculty of Dentistry for dental treatment to evaluate self-reported xerostomia symptoms and reveal the relationships between sociodemographic variables and xerostomia in different age groups.

This article was previously published as a pre-print on the Authorea website on June 2, 2024.

## Materials and methods

Data collection

This cross-sectional study was approved by the Clinical Research Ethics Committee of the Ankara University Faculty of Dentistry in accordance with the Declaration of Helsinki (approval number: 14/02; date: 09.12.2020) and adhered to STROBE (Strengthening the Reporting of Observational Studies in Epidemiology) guidelines. At the beginning of the study, patients between the ages of 20 and 65 years who had contact information in the patient registration system were screened by their gender. In a preliminary phone call, information about the study was provided and 300 volunteer participants were included, which was close to the sample size previously determined to be 266. The interviews continued until the number of male and female participants was equal. Patients with insufficient reading, comprehension, and answering skills and those who did not use WhatsApp were excluded from the study. Later, the surveys prepared on Google Forms were sent via WhatsApp between January and August 2021.

Consent was obtained from all volunteers by reading the consent form for the study and clicking on the consent tab. After this stage, they were able to start the survey. The survey settings required the previous question to be completed before proceeding to the next question. Complete surveys were those found to be suitable for statistical analysis and were included in the study. Each participant filled out the survey form without any intervention from the researcher.

Survey form

The survey consisted of two parts. The first part of the survey included sociodemographic information, such as first and last name, phone number, gender, date of birth, age, medical history, and medication use, as reported by the patients. In addition, smoking habits, alcohol consumption, the use of removable prostheses, and whether the users were satisfied with the retention of their prostheses were inquired about in this section.

The second part of the survey was about xerostomia which was modified from an inventory used in a previous study (presented in Appendices) [1]. The presence and severity of xerostomia was measured using XI, which consists of 11 questions. The questions were scored according to a five-point, one-way scale of ratings ranging from “never” to “always.” They were scored as never = 0, hardly ever = 1, occasionally = 2, frequently = 3, and always = 4. The responses of each patient were scored and added to achieve a single XI score [18,23]. The scores ranged from 11 (no xerostomia) to 44 (severe xerostomia), with high scores indicating signs of severe xerostomia. The value ranges for each degree were as follows: no dry mouth (0-11 points), mild dry mouth (12-22 points), medium dry mouth (23-33 points), and severe dry mouth (34-44 points) [23]. Based on a previous study, we added three more questions to the XI (presented in Appendices) [11]. The other symptoms of xerostomia, including recurring mouth sores, self-reported halitosis, and burning in the mouth, were inquired about using a five-point scale as follows: “never,” “hardly ever,” “occasionally,” “frequently,” and “always.” No preliminary studies were conducted, as the validity and reliability of the inventory forms used were proven in previous studies [19].

Data analysis

In a power calculation, 266 patients were needed in the study for a 95% confidence interval with a 5% margin of error. The sample size was determined to be 266 according to a G*power analysis with a 95% confidence level and a 5% margin of error. The effect level in the power analysis was established as 0.20 before the study to determine the size of the sample; the α value was 0.05, and the power value (1-β) was 0.90. Accordingly, the necessary sample size was determined to be 266 subjects. A total of 300 patients, aged 20-65 years, who applied to the Faculty of Dentistry for treatment before the pandemic were included to ensure the homogeneous distribution and accurate collection of the data. There were 150 female and 150 male patients, with an equal number in all age groups. Microsoft Excel (Microsoft Corp., Redmond, WA, USA) and SPSS Statistics for Windows, Version 24 (IBM Corp., Armonk, NY, USA) were employed for statistical analysis. A p-value <0.05 was interpreted as statistically significant. Descriptive statistics were performed based on the median value. The relationships between the presence of xerostomia and other variables were analyzed using the chi-square test. These categorical variables included sociodemographic characteristics such as age, gender, the presence of a systemic disease, the use of medications, smoking, alcohol consumption, and the use of removable prostheses. The differences in the xerostomia score according to the other variables were analyzed using a t-test and analysis of variance (ANOVA). Normality was ensured because the scores were in accordance with the normal distribution, and therefore, parametric test techniques were used in analyses.

## Results

Sociodemographic data

This research population consisted of 300 patients. Age, gender, the presence of a systemic disease, medication use, smoking, alcohol consumption, and the use of removable prostheses were evaluated as sociodemographic data. There were five age groups, i.e., 20-29, 30-39, 40-49, 50-59, and 60-65 years, with 60 (20.0%) patients in each age group. Each age group consisted of 30 (50.0%) female and 30 (50.0%) male patients. The ages of the patients ranged from 20 to 65 years, with an average of 43.86 ± 13.92 years. The mean age of the female patients was 43.70 ± 13.90 years, and the mean age of the male patients was 44.02 ± 13.98 years. Table 1 summarizes the demographic characteristics of the study participants. The majority did not have any systemic disease (209, 69.7%), and were not on medications (206, 68.7%). Overall, 103 (36.4%) patients reported that they smoked cigarettes. Of the patients who smoked, 25 (31.1%) reported that they smoked between zero and five cigarettes per day, 31 (35.9%) smoked between six and 15 cigarettes per day, and 28 (33.0%) smoked more than 15 cigarettes per day.

**Table 1:** Analysis of sociodemographic data (N = 300). Data are presented as the number of patients (n) and percentages (%).

Characteristics		n	%
Age (years)	20–29	60	20.0
	30–39	60	20.0
	40–49	60	20.0
	50–59	60	20.0
	60–65	60	20.0
Gender	Female	150	50.0
	Male	150	50.0
Do you have a systemic disease?	Yes	91	30.3
	No	209	69.7
Do you take any medications?	Yes	94	31.3
	No	206	68.7
If yes, how many?	1	52	54.7
	2–3	31	32.6
	More than 3	12	12.6
Do you smoke?	Yes	103	36.4
	No	197	65.7
Cigarettes per day	0–5	25	31.1
	6–15	31	35.9
	More than 15	28	33.0
Do you consume alcohol?	Yes	56	18.7
	No	244	81.3
Do you use removable prostheses?	Yes	22	7.3
	No	278	92.7
If you use removable prostheses, are you satisfied with the retention of your prostheses?	Yes	11	50.0
	No	11	50.0

XI and measurement of xerostomia symptoms

**Figure 1:**
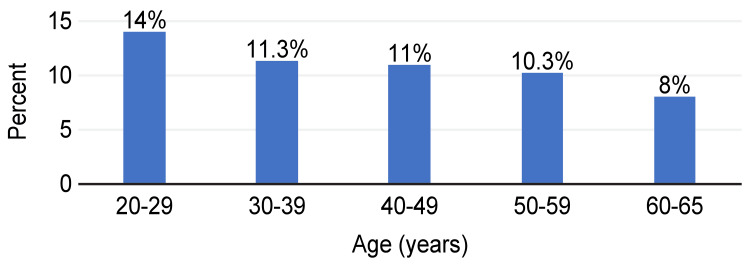
Presence of xerostomia by age groups.

The presence of xerostomia was 54.6% (164 patients). It was 14.0% (42 patients) in the 20-29-year age group, 11.3% (34 patients) in the 30-39-year age group, 11% (33 patients) in the 40-49-year age group, 10.3% (31 patients) in the 50-59-year age group, and 8% (24 patients) in the 60-65-year age group (Figure 1).

Table 2 shows the relationship between the presence of xerostomia and age group. There was a significant relationship between age and xerostomia (p = 0.023). The presence of xerostomia decreased as age increased. The presence of xerostomia was 30.0% (90 patients) among female patients and 24.6% (74 patients) among male patients. The relationship between xerostomia and gender was examined, and a significant relationship was found (p = 0.028). The presence of xerostomia was higher among the female patients. As seen in Table 2, the presence of xerostomia in patients with a systemic disease was 59.4% (54 patients), and it was 52.6% (110 patients) among patients with no systemic disease. The relationship between the presence of systemic disease and xerostomia was examined, and no significant relationship was found (p = 0.549). There was no significant relationship between the presence of xerostomia and the use of medication.

**Table 2:** Evaluation of the relationships between the presence of xerostomia and other variables (N = 300). *: p < 0.05; Pearson’s chi-square test. Data are presented as the number of patients (n) and percentages (%).

		Xerostomia					
		None		Yes		Chi-squared	P-value
		n	%	n	%		
Age (years)	20–29	18	30.0	42	70.0	17.813	0.023
	30–39	26	43.3	34	56.6		
	40–49	27	45.0	33	55.0		
	50–59	29	48.3	31	59.9		
	60–65	36	60.0	24	40.0		
Gender	Female	60	40.0	90	60.0	7.132	0.028
	Male	76	50.7	74	49.3		
Do you have a systemic disease?	Yes	37	40.7	54	59.4	1.198	0.549
	No	99	47.4	110	52.6		
Do you take any medications?	Yes	37	39.4	57	60.6	1.970	0.373
	No	99	48.1	107	51.9		
Do you smoke?	Yes	52	50.5	51	49.5	3.633	0.163
	No	84	42.6	113	57.4		
Cigarettes per day	0–5	10	31.3	22	68.7	10.015	0.04*
	6–15	25	67.6	12	32.4		
	More than 15	17	50.0	17	50.0		
Do you consume alcohol?	Yes	23	41.1	33	58.9	0.862	0.65
	No	113	46.3	131	53.6		
Do you use removable prostheses?	Yes	14	63.6	8	36.4	4.007	0.135
	No	122	43.9	156	56.1		

There was a significant relationship between the number of cigarettes smoked per day and xerostomia (p = 0.04) (Table 2). Overall, 49.5% (51 patients) of individuals who smoked were observed to have xerostomia. The presence of xerostomia was 68.7% (22 patients) among patients who smoked zero to five cigarettes per day, 32.4% (12 patients) among the patients who smoked six to 15 cigarettes per day, and 50.0% (17 patients) among patients who smoked more than 15 cigarettes per day. Among patients who consumed alcohol, 58.9% (33 patients) reported xerostomia. No significant relationship was found between xerostomia and alcohol consumption (p = 0.65). Xerostomia was observed in 36.4% (8 patients) of patients who used removable prostheses. No significant relationship was found between xerostomia and the use of removable prostheses (p = 0.135) (Table 2).

When the symptoms of xerostomia on the XI were examined, the highest mean score was obtained for the question, “Do you feel dryness in your skin?.” This was followed by “Do you feel dryness on your lips?” and “Do you wake up and drink water at night?.” The questions with the lowest mean scores were “Do you use candies or throat drops to relieve your dry mouth?,” “Do you feel your mouth becomes dry while eating?,” and “Do you have difficulty swallowing any food?” (Table 3).

**Table 3:** Evaluation of symptoms of xerostomia (N = 300). Data are presented as the number of patients (n) and percentages (%). SD = standard deviation

Symptoms of xerostomia	1.0		2.0		3.0		4.0		5.0		Mean	SD	Median
	n	%	n	%	n	%	n	%	n	%			
Do you need a drink to swallow food?	96	32.0	70	23.3	117	39.0	11	3.7	6	2.0	2.20	1.00	2.00
Do you feel your mouth becomes dry while eating?	129	43.0	92	30.7	74	24.7	5	1.7	0	0.0	1.85	0.85	2.00
Do you wake up and drink water at night?	74	24.7	67	22.3	120	40.0	24	8.0	15	5.0	2.46	1.10	3.00
Do you feel dryness in your mouth?	103	34.3	76	25.3	97	32.3	19	6.3	5	1.7	2.16	1.02	2.00
Do you have difficulty swallowing dry food?	103	34.3	83	27.7	98	32.7	13	4.3	3	1.0	2.10	0.96	2.00
Do you use candies or throat drops to relieve your dry mouth?	191	63.7	78	26.0	30	10.0	1	0.3	0	0.0	1.47	0.69	1.00
Do you have difficulty swallowing any food?	132	44.0	85	28.3	79	26.3	4	1.3	0	0.0	1.85	0.86	2.00
Do you feel dryness in your skin?	72	24.0	55	18.3	112	37.3	39	13.0	22	7.3	2.61	1.19	3.00
Do you feel dryness in your eyes?	104	34.7	79	26.3	79	26.3	26	8.7	12	4.0	2.21	1.13	2.00
Do you feel dryness on your lips?	67	22.3	52	17.3	133	44.3	36	12.0	12	4.0	2.58	1.08	3.00
Do you feel dryness inside your nose?	71	23.7	75	25.0	116	38.7	28	9.3	10	3.3	2.44	1.01	3.00

In this study, face-to-face interviews with patients and a face-to-face oral examination could not be performed due to restrictions from the COVID-19 pandemic. Therefore, self-reported halitosis, self-reported burning mouth, and self-reported recurrent mouth sores with xerostomia symptoms were evaluated, as shown in Table 4. According to the evaluation, the symptom with the highest mean score in the entire population was halitosis, followed by recurrent mouth sores and burning mouth, according to the mean score. When these symptoms were evaluated in patients with xerostomia, the symptom with the highest mean score was halitosis, and the scores were similar to the halitosis scores in the entire study population. In patients with xerostomia, halitosis was followed by recurring mouth sores and burning mouth, according to the mean score (Table 4).

**Table 4:** Evaluation of other symptoms of xerostomia (N = 300). Data are presented as the number of patients (n) and percentages (%). SD = standard deviation

Other symptoms of xerostomia	1.0		2.0		3.0		4.0		5.0		Mean	SD	Median
	n	%	n	%	n	%	n	%	n	%			
Do you ever have mouth sores?	86	28.7	66	22.0	121	40.3	22	7.3	5	1.7	2.31	1.02	3.00
Do you feel burning in your mouth?	154	51.3	100	33.3	37	12.3	7	2.3	2	0.7	1.68	0.83	2.00
Do you have halitosis?	79	23.3	74	24.7	112	37.3	27	9.0	17	5.7	2.49	1.11	4.00

The XI scores were examined according to age using ANOVA (Table 5). There were statistically significant differences between different age groups according to the XI (p = 0.001). The majority of subjects were affected by xerostomia and this condition was common among the 20-29-year age group.

**Table 5:** Examination of Xerostomia Inventory scores in terms of age. *: p < 0.05, analysis of variance test. Data are presented as the number of patients (n). SD = standard deviation

	Age (years)	N	Mean	SD	Median	F	P-value
Xerostomia Inventory	20–29	60	15.52	7.27	15.00	4.567	0.001 *
	30–39	60	13.65	6.59	13.00		
	40–49	60	12.82	6.10	13.00		
	50–59	60	12.10	7.06	12.50		
	60–65	60	10.58	6.09	11.00		

The XI scores were examined according to gender using a t-test (Table 6). There was a statistically significant difference between the female and male patients in terms of xerostomia (p = 0.004). According to the results, the mean scores of the female patients were higher.

**Table 6:** Examination of Xerostomia Inventory scores in terms of gender (N = 300). *: p < 0.05, t-test. Data are presented as the number of patients (n). SD = standard deviation

	Gender	N	Mean	SD	Median	t	P-value
Xerostomia Inventory	Female	150	14.05	7.28	14.00	2.890	0.004 *
	Male	150	11.81	6.10	11.00		

## Discussion

The results of the current study reveal the severity and presence of xerostomia and self-reported symptoms in an adult Turkish patient population aged 18 years and over. Xerostomia affected one out of every two people and was more common in the 20-29-year age group. The prevalence of xerostomia among adults in New Zealand and Australia was found to be lower than these results. Those studies reported that xerostomia affected one out of eight people [20,21]. However, the age range of those study populations was 18-75 years. Regarding age groups, xerostomia was common in the younger age group, which is similar to these results. Differences in the prevalence of xerostomia may be due to geographical location or the instrument used for xerostomia research. The use of a multiple-choice comprehensive survey such as the XI in this study, instead of a one-question survey such as “How often does your mouth feel dry?” could explain the high prevalence rates. An example of a study with a high prevalence of xerostomia was a survey conducted in 2020 among 75 dentistry students over the age of 18 using XI. The participants in that study did not have systemic diseases and did not use medications (53, 73.6%) [17]. Additionally, xerostomia was categorized as “sometimes,” “often,” and “always,” which was similar to the present study, and this increased the prevalence of xerostomia to 60% (586 participants) [24]. However, using only one standard question, the prevalence of xerostomia was reported as 51.4% among a population of 3,313 individuals between the ages of 20 and 80 years [10]. Similarly, the presence of xerostomia was 54.6% (164 patients) in the present study. Even with the use of different xerostomia tools, the high prevalence rates ​​may be due to geographical differences.

In the present study, the higher presence of xerostomia among female patients was statistically significant (p = 0.028), and this result is in line with the results of many previous cross-sectional studies that found that the prevalence of xerostomia to be higher among female patients compared to male patients [14,20,23]. However, the factors that cause xerostomia in women cannot be explained by the present study.

Studies have suggested that xerostomia increases with age and is caused by medication use in population groups over 65 years old [4,6,12]. However, in this population, the age range was 20-65 years and xerostomia did not increase with increasing age (p = 0.028). It was also not caused by medication use (p = 0.373). The increase in the prevalence of xerostomia with age is attributed to the use of a wide variety of medications by the elderly [10,25]. However; there was a difference in xerostomia values ​​between the age groups in this population (p = 0.001).

The reason why “dry lips” and “dry skin” were included in the survey questions in the present study was to catch diseases such as Sjögren’s syndrome, but there was no such patient group in this population. Clinicians should be familiar with the symptoms of the disease and be prepared to take an active role in the diagnosis, management, and treatment of oral complications. When the symptoms of xerostomia, including halitosis, burning mouth, and recurring mouth sores, were examined, self-reported halitosis had the highest mean score. These three symptoms of xerostomia were evaluated in a previous study, which reported that halitosis was more common compared to the other symptoms [4]. Similar to this study, halitosis was followed by recurring mouth sores and burning in the mouth.

In this study, the presence of xerostomia increased as the number of cigarettes used increased in younger age groups. Xerostomia is seen in one of out every two smokers (51, 49.5%). Similarly, in a study conducted in 2022, this rate was reported as 48.7% (19 participants) [26]. In a meta-analysis investigating the relationship between smoking and xerostomia prevalence, the prevalence of smoking-related xerostomia was generally between 8.3% and 60%, and different results were mentioned according to geographical location and age groups. In addition, it has been reported that smoking may result in xerostomia in young people [27].

When smoking was evaluated according to the number of daily cigarettes smoked in the present study, a significant relationship was observed between xerostomia and smoking (p = 0.004). Reports in the literature indicate that xerostomia is observed in about one-third of individuals who smoke six to 15 cigarettes per day (12, 32.4%) and in about half of individuals who smoke more than 15 cigarettes per day (17, 50.0%). In other studies, the relationship between xerostomia and the number of cigarettes smoked per day has not been investigated. However, the percentage of people who smoked a high number of cigarettes was higher in this population compared to other studies. In a study conducted in Sweden, no significant relationship was found between smoking and xerostomia [11]. While the rate of individuals who smoked between six and 15 cigarettes per day was 24.2% (39 participants) in the latter study, this percentage was higher at 35.9% (31 patients) in the present study. The relationship between the prevalence of xerostomia and the number of cigarettes smoked per day has not been determined in other studies as the smoking rates were not high, and this relationship has not been specifically investigated. This should be investigated in future studies. This study showed that the presence of both xerostomia and smoking increased as age decreased, albeit not statistically significantly (p = 0.079). Contrary to the literature, the decrease in xerostomia with the decrease in age may be due to the increased rate of smoking as age decreased in this population.

As the percentage of alcohol consumers was low in this population (56, 18.7%), no statistically significant relationship was found between the presence of xerostomia and alcohol consumption (p = 0.65). The number of studies evaluating the relationship between alcohol and xerostomia is limited in the literature. Alcohol consumption severely affects mouth sensations such as thirst and xerostomia [28]. The consumption of alcoholic beverages has been reported to reduce the secretion of saliva [29]. In this study, xerostomia was observed in approximately one in two (33, 58.9%) alcohol consumers, suggesting that alcohol consumption increased xerostomia, as indicated in the literature.

Due to the low number of patients using prostheses in this population (22, 7.3%), no significant result could be obtained in the relationship between xerostomia and the use of removable prostheses (p = 0.135). Nevertheless, the determination of xerostomia in one-third (8, 36.4%) of patients who used removable prostheses was significant. Similarly, the prevalence of xerostomia was determined to be 34.9% (80 patients) in a study, which was conducted through questionnaires administered to a Turkish population who used removable prostheses [30].

The strength of this cross-sectional study was the limited use of drugs in the young population in the sample. Additionally, the reason for the high prevalence was daily smoking which was significantly higher in young people, confirming that smoking also affected the presence of xerostomia with questionnaires.

This study had several limitations. As this study was conducted during the COVID-19 pandemic, the inability to perform any examinations on the patients constitutes the limitation of the study. While perception is important to understand, clinical validation may be required to confirm symptoms and determine the severity of symptoms. More reliable results can be obtained if screening with oral examination is performed in larger populations in further studies. In addition, it is necessary to evaluate the psychological factors that increased with the pandemic and are among the etiological factors of xerostomia. The inability to collect saliva from patients during this period prevented us from evaluating the relationship between hyposalivation and xerostomia among Turkish patients. However, ours is the first study using surveys of xerostomia alone among patients who applied to the hospital for dental care. Some bias must be considered as the studied population consisted of individuals who previously applied to our faculty for dental treatment, as xerostomia is known to cause oral and dental problems. Additionally, excluding patients with inadequate reading, understanding, and answering skills and who do not use WhatsApp can be a bias of the study. It can be said that patients with higher education levels and access to information on the internet are reflected in the study population.

## Conclusions

This study sheds light on the current status, symptoms, and etiology of xerostomia presence in the young population in Turkey. While factors such as gender, age, and smoking are associated with xerostomia, knowledge gaps regarding medication, alcohol, and prosthesis use continue. The findings highlight that smoking affects the presence of xerostomia in the young population and highlight the need for public health interventions to raise awareness of its potential risks. Healthcare providers can play an important role in improvement by increasing awareness and implementing evidence-based strategies. Such efforts are vital to addressing the growing public health problems caused by xerostomia. Additionally, future studies with clinical examinations would be beneficial to corroborate these findings.

## Appendices

**Table 7:** Xerostomia Inventory (XI-11) and the other symptoms of xerostomia. While the first 11 questions are included in the Xerostomia Inventory, the 12th, 13th, and 14th questions are about other symptoms of xerostomia. Example responses: (1) For items that are “never,” please circle A. (2) For items that are “hardly ever,” please circle B. (3) For items that are“occasionally,” please circle C. (4) For items that are “frequently,” please circle D. (5) For items that are “always,” please circle E. 0: never; 1: hardly ever; 2: occasionally; 3: frequently; 4: always

Items	Responses				
	0	1	2	3	4
Q1. Do you need a drink to swallow food?	(A) Never	(B) Hardly ever	(C) Occasionally	(D) Frequently	(E) Always
Q2. Do you feel your mouth dry while eating?	(A) Never	(B) Hardly ever	(C) Occasionally	(D) Frequently	(E) Always
Q3. Do you wake up and drink water at night?	(A) Never	(B) Hardly ever	(C) Occasionally	(D) Frequently	(E) Always
Q4. Do you feel dryness in your mouth?	(A) Never	(B) Hardly ever	(C) Occasionally	(D) Frequently	(E) Always
Q5. Do you have difficulty swallowing dry food?	(A) Never	(B) Hardly ever	(C) Occasionally	(D) Frequently	(E) Always
Q6. Do you use candies or throat drops to relieve your dry mouth?	(A) Never	(B) Hardly ever	(C) Occasionally	(D) Frequently	(E) Always
Q7. Do you have difficulty swallowing any food?	(A) Never	(B) Hardly ever	(C) Occasionally	(D) Frequently	(E) Always
Q8. Do you feel dryness in your skin?	(A) Never	(B) Hardly ever	(C) Occasionally	(D) Frequently	(E) Always
Q9. Do you feel dryness in your eyes?	(A) Never	(B) Hardly ever	(C) Occasionally	(D) Frequently	(E) Always
Q10. Do you feel dryness in your lips?	(A) Never	(B) Hardly ever	(C) Occasionally	(D) Frequently	(E) Always
Q11. Do you feel dryness inside your nose?	(A) Never	(B) Hardly ever	(C) Occasionally	(D) Frequently	(E) Always
Q12. Do you ever have any mouth sores?	(A) Never	(B) Hardly ever	(C) Occasionally	(D) Frequently	(E) Always
Q13. Do you feel burning in your mouth?	(A) Never	(B) Hardly ever	(C) Occasionally	(D) Frequently	(E) Always
Q14. Do you have halitosis?	(A) Never	(B) Hardly ever	(C) Occasionally	(D) Frequently	(E) Always
